# Cell-free ascites from ovarian cancer patients induces Warburg metabolism and cell proliferation through TGFβ-ERK signaling

**DOI:** 10.1007/s11357-023-01056-1

**Published:** 2024-01-10

**Authors:** Dóra Szeőcs, Beáta Vida, Gábor Petővári, Szilárd Póliska, Eszter Janka, Adrienn Sipos, Karen Uray, Anna Sebestyén, Zoárd Krasznai, Péter Bai

**Affiliations:** 1 Department of Medical Chemistry, Faculty of Medicine, University of Debrecen, Debrecen, Hungary 4032; 2 Center of Excellence, The Hungarian Academy of Sciences, Debrecen, Hungary; 3 Department of Obstetrics and Gynecology, Faculty of Medicine, University of Debrecen, Debrecen, Hungary 4032; 4 Department of Pathology, Semmelweis University, Budapest, Hungary; 5 Department of Biochemistry and Molecular Biology, Faculty of Medicine, University of Debrecen, Debrecen, Hungary 4032; 6 Department of Dermatology, Faculty of Medicine, University of Debrecen, Debrecen, Hungary 4032; 7 HUN-REN-DE Cell Biology and Signaling Research Group, Debrecen, Hungary 4032; 8 MTA-DE Lendület Laboratory of Cellular Metabolism, Debrecen, Hungary 4032; 9 Research Center for Molecular Medicine, Faculty of Medicine, University of Debrecen, Debrecen, Hungary 4032

**Keywords:** Ovarian cancer, Ascites, Proliferation, Warburg metabolism, Coupled respiration, Glucose oxidation, Fatty acid oxidation, Tumor microenvironment

## Abstract

Ascites plays a key role in supporting the metastatic potential of ovarian cancer cells. Shear stress and carry-over of cancer cells by ascites flow support carcinogenesis and metastasis formation. In addition, soluble factors may participate in the procarcinogenic effects of ascites in ovarian cancer. This study aimed to determine the biological effects of cell-free ascites on carcinogenesis in ovarian cancer cells. Cell-free ascites from ovarian cancer patients (ASC) non-selectively induced cell proliferation in multiple models of ovarian cancer and untransformed primary human dermal fibroblasts. Furthermore, ASC induced a Warburg-type rearrangement of cellular metabolism in A2780 ovarian cancer cells characterized by increases in cellular oxygen consumption and glycolytic flux; increases in glycolytic flux were dominant. ASC induced mitochondrial uncoupling and fundamentally reduced fatty acid oxidation. Ascites-elicited effects were uniform among ascites specimens. ASC-elicited transcriptomic changes in A2780 ovarian cancer cells included induction of the TGFβ-ERK/MEK pathway, which plays a key role in inducing cell proliferation and oncometabolism. ASC-induced gene expression changes, as well as the overexpression of members of the TGFβ signaling system, were associated with poor survival in ovarian cancer patients. We provided evidence that the activation of the autocrine/paracrine of TGFβ signaling system may be present in bladder urothelial carcinoma and stomach adenocarcinoma. Database analysis suggests that the TGFβ system may feed forward bladder urothelial carcinoma and stomach adenocarcinoma. Soluble components of ASC support the progression of ovarian cancer. These results suggest that reducing ascites production may play an essential role in the treatment of ovarian cancer by inhibiting the progression and reducing the severity of the disease.

**Supplementary Information:**

The online version contains supplementary material available at 10.1007/s11357-023-01056-1.

## Background

Ovarian cancer is the second most common gynecological malignancy and is characterized by poor clinical outcomes [1, 2]. The incidence of ovarian cancer peaks in the older age, in the UK is the highest among the 75–79 years age spread [3], similar to the USA, where the highest rate of incidence is among the 55–64 years age spread and the 65–74 years age spread [4]. Elderly patients (≥ 65 years of age) usually present with higher grade tumors as compared to younger patients [5]. Most deaths from ovarian cancer also peak among the elderly [4, 6], and younger patients have advantage over elderly patients in terms of survival [7]. Taken together, ovarian cancer is tightly associated with the aging process and with biological age.

Ascites, formed in the peritoneal cavity, is an important constituent of the ovarian cancer tumor microenvironment. Ascites has a very complex composition that includes cytokines and inflammatory regulators [8–11], adipokines [12–14], extracellular vesicles [15–18], transforming growth factor beta (TGFβ)-induced [19], vascular endothelial growth factor [13], growth factor receptor ligands [20], factors regulating cellular adhesion [8], metabolites [8], cholesterol [21, 22], bacteria, and bacterial metabolites (reviewed in [23]). Ascites supports transcoelomic metastasis formation (dissemination) of the primary tumor in the peritoneum. Furthermore, ascites can interfere with therapeutic responses [8].

According to the current understanding, the prometastatic and procarcinogenic effects of ascites fluid are primarily associated with shear stress and increased intraabdominal pressure [8]. Nevertheless, the non-cellular molecular constituents of ascites [24] possess potential biological activity that can potentially impact cancer progression or metastasis formation. In this study, we set out to decipher the biological effects of cell-free ascites from advanced-stage ovarian cancer patients in ovarian cancer cell models.

## Results

### Cell-free ascites induces cell proliferation in ovarian cancer cells and primary fibroblasts

**Fig. 1:**
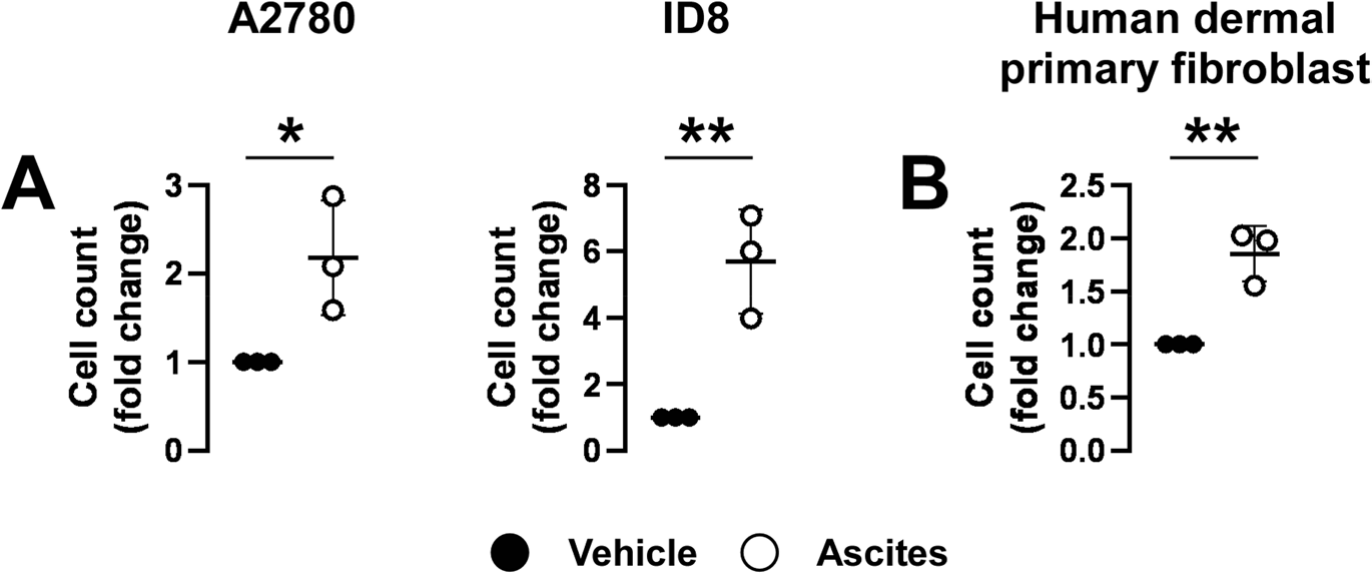
Cell-free ascites induces cell proliferation in ovarian cancer cells and primary fibroblasts. **A** A2780 (1000 cells/well) or ID8 cells (1000 cells/well) were seeded into 96-well plates and 10% of the media volume was replaced with ascites or PBS (vehicle) for 48 h. Cell numbers were assessed using sulforhodamine B (SRB) assays. **B** Primary human dermal fibroblasts (3000 cells/well) were seeded into 96-well plates, and 10% of the media volume was replaced with ascites or PBS (vehicle) for 48 h. Cell numbers were assessed using the SRB assay after the proliferation period. The experiment was performed once using the three ascites samples. Cell numbers were normalized to the control. Vehicle and ascites-treated cells were compared using *t*-tests. * and ** indicate statistical significance between vehicle and ascistes-treated cells at *p* < 0.05 or *p* < 0.01, respectively

Ovarian cancer cells were treated with cell-free ascites fluid from three unrelated advanced-stage ovarian cancer patients. Ascites induced cell proliferation in two different models of ovarian cancer (A2780 and ID8) (Fig. 1A). To determine if the effects of cell-free ascites fluid are specific to ovarian cancer cells, we treated primary human dermal fibroblasts with cell-free ascites. Ascites from ovarian cancer patients induced cell proliferation in fibroblasts to a similar extent as in the ovarian cancer cell models (Fig. 1B).

### Ascites treatment supports the proliferation of A2780 ovarian cancer cells through TGFβ-ERK/MEK activation

**Fig. 2:**
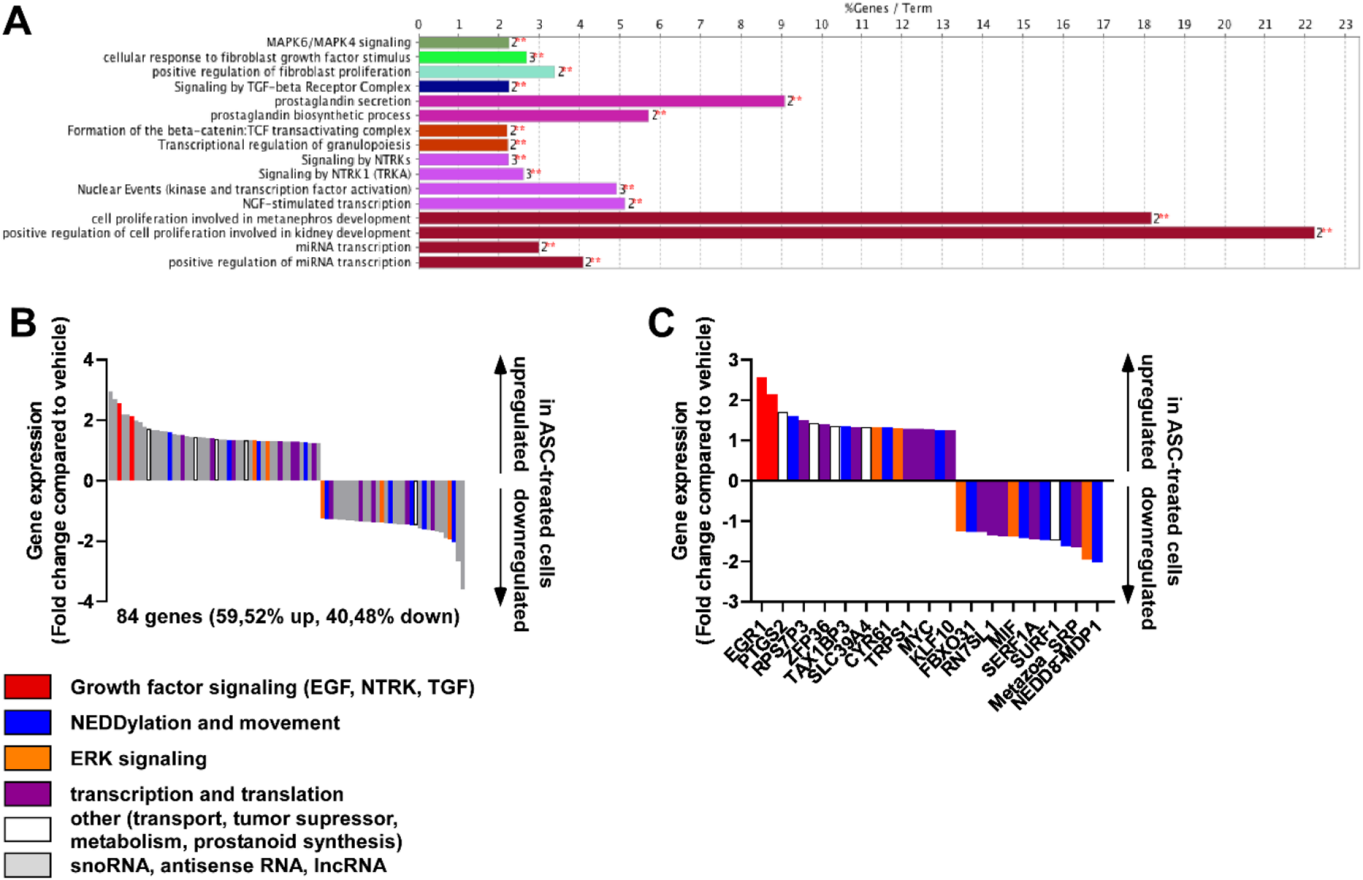
Ascites treatment induces pro-proliferation and survival pathways. RNA sequencing was performed on A2780 cells treated with cell-free ascites for 2 h. **A** Sequences were used to probe the GO database to assess gene function. **B**,** C** Gene function was assessed through a literature search. All transcripts are depicted in **B**, while non-coding RNAs were omitted in **C**. Abbreviation: ASC, ascites

To understand the mechanism for the effects of ascites, we performed RNA sequencing in cells treated with ascites for 2 h. Each ascites sample was applied to a separate pool of A2780 cells. A comparison of the transcriptomes of vehicle-treated cells versus ascites-treated cells revealed the differential expression of 84 genes; 59.52% of genes were upregulated and 40.48% were downregulated (Fig. 2A**, Supplementary Table 1**). The biggest changes to mRNA transcripts quantities were 2^3^ for the upregulated and 2^−3^ fold for the downregulated genes.

To decipher the functional changes associated with ascites-elicited gene expression changes, we performed two unbiased in silico analyses by mapping the transcriptome using the KEGG database and the Reactome database (Fig. 2A**, **Supplementary Table 2, 3; Supplementary Figs. 1, 2). In addition, we performed a literature search to better understand the functions of the identified genes. Most of the genes identified were sno/lnc/antisense RNAs that we eliminated from further analysis (Fig. 2B, C). Both analyses highlighted the influence of ascites treatment on the expression of multiple genes linked to mitogen-activated protein kinases (MEKs), extracellular-signal-regulated kinases (ERKs), elements of growth factors signaling encompassing epidermal growth factor, neurotrophic tyrosine receptor kinase, and TGF signaling. Genes associated with NEDDylation, movement, transcription, and translation were also identified; however, these genes were not specific for signaling pathways and, hence, were not useful for identifying ascites-elicited functional changes.

**Fig. 3:**
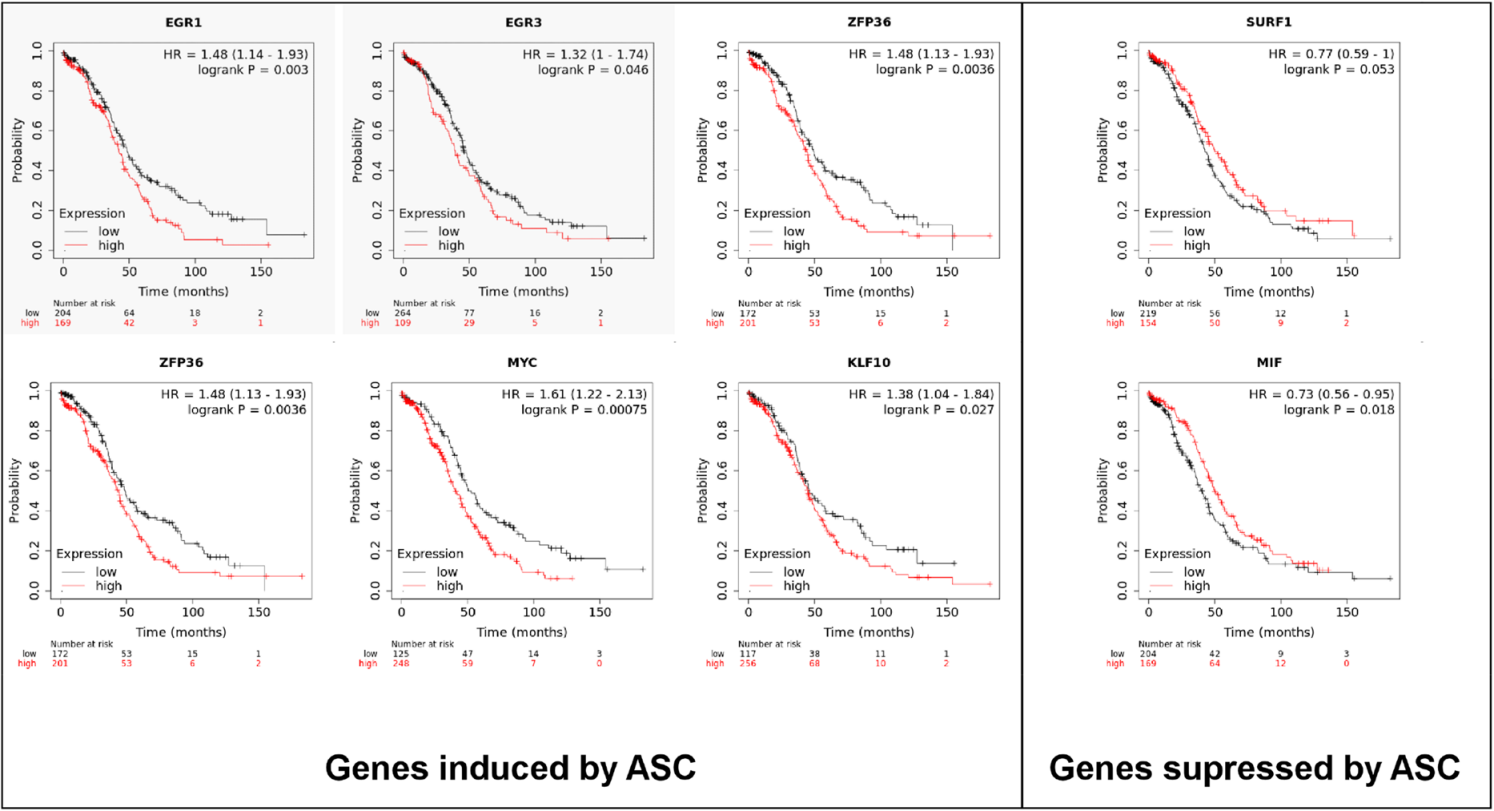
Ascites-induced gene expression changes associate with shorter patient survival. The genes identified in RNA sequencing were accessed in the kmplot.com database. The RNA sequencing database was used. (Accessed: 2022. 09. 02) Abbreviation: ASC, ascites

The influence of these expression changes on patient survival in humans was assessed. To that end, survival data of ovarian cancer patients was assessed using the kmplot.com database [25]. Importantly, tumor overexpression of a set of genes induced by ascites treatment was associated with shorter patient survival (Fig. 3), and, vice versa, the tumor overexpression of a set of genes that were suppressed by ascites treatment was associated with better patient survival (Fig. 3). These data suggest that ascites treatment-associated gene expression changes cumulatively shorten patient survival in ovarian cancer patients.

**Fig. 4:**
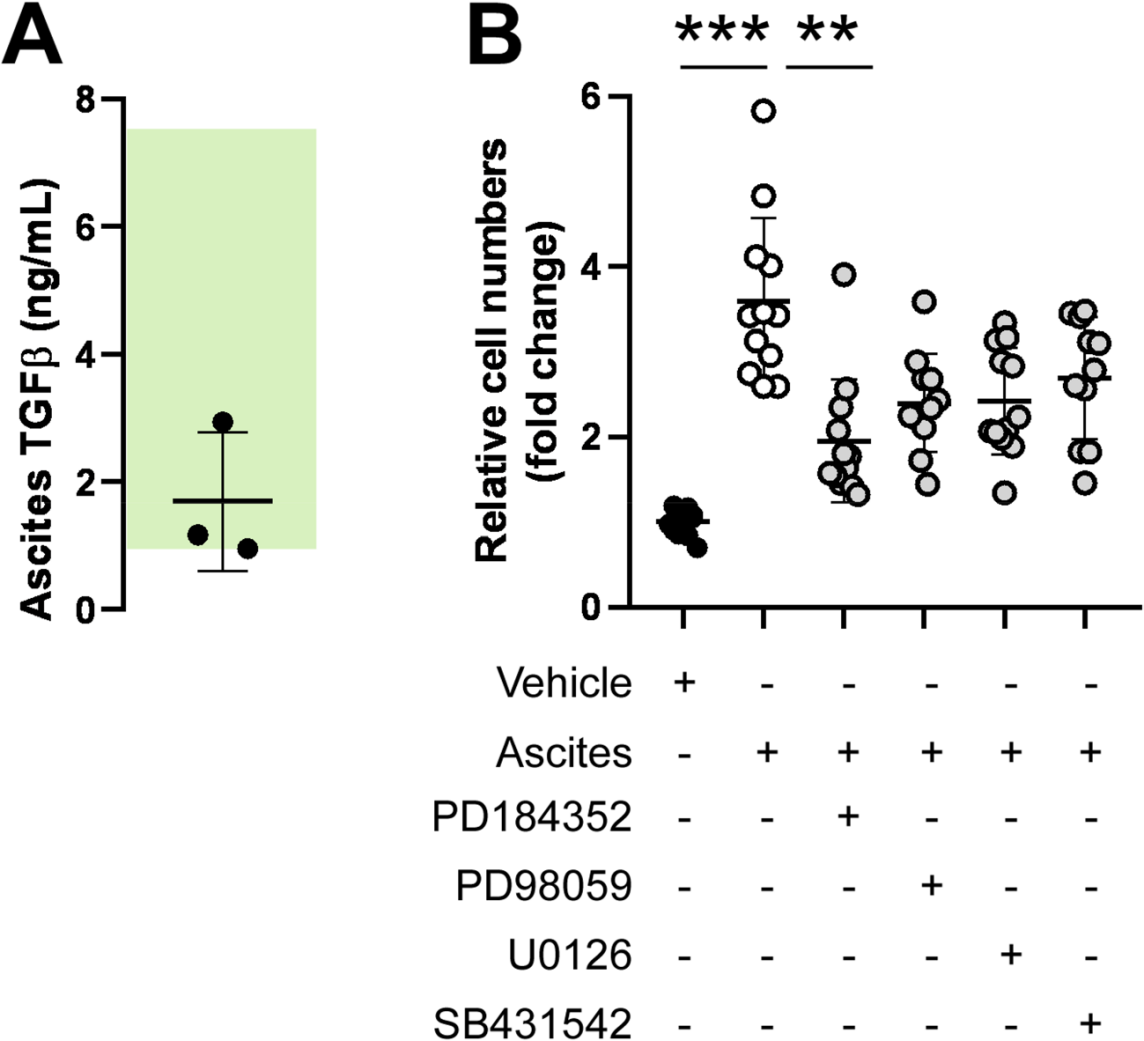
ASC treatment induces the TGFβ-ERK/MEK pathway, supporting ovarian cancer cell proliferation and shortening patient survival. **A** TGFβ levels were measured in the ascites fluid by ELISA. The green rectangle shows the total serum TGFβ concentration based on the literature in non-diseased individuals. The experiment was performed once using the three ascites samples. **B** A2780 cells (1 000 cells/well) were treated with PD184352 (20 µM), PD98059 (20 µM), U0126 (20 µM), or SB432542 (10 µM) with or without 10% ascites for 48 h. Cell numbers were assessed using the SRB assay after the proliferation period. PBS was used as a vehicle. The experiment was performed four times using the three ascites samples. Cell numbers were normalized to the control. Experimental groups were compared using Kruskal–Wallis followed by the Dunn post hoc test. ** and *** symbolize statistical significance between the indicated groups at *p* < 0.01 or *p* < 0.001, respectively

The RNA sequencing experiments suggested that ascites treatment in A2780 cells activated TGFβ-ERK/MEK signaling. Therefore, the elements of this signaling pathway were assessed. TGFβ, ranging from 0.96 to 2.94 ng/mL, was detected in all ascites samples (individual values: patient 1: 1.17 ng/mL; patient 2: 2.94 ng/mL; patient 3: 0.96 ng/mL) (Fig. 4A). To link the activation of the TGFβ-ERK/MEK pathway to ASC-elicited effects, cells were treated with the MEK inhibitors, PD184352, PD98059, and U0126, and an inhibitor of TGFβ signaling, SB432542. Ascites-induced hyperproliferation was blocked by PD184352. Treatment with PD98059, U0126, and SB432542 also reduced ascites-induced cell proliferation, but the differences were not significant (Fig. 4B). In addition to that, treatment of A2780 cells with TGFβ induced similar gene expression changes as ascites treatment (Fig. 4C). These results suggest that ascites-mediated induction of TGFβ, ERK, and MEK-related genes support the proliferation of ovarian cancer cells.

### Treatment of ovarian cancer cells with ascites induces Warburg-type rearrangement of oxidative metabolism

Rearrangement of cellular metabolism can support cancer cell proliferation [26, 27]. Warburg rearrangement of metabolism occurs in ovarian cancer cells [28–31]. Furthermore, MEK and ERK activation was linked to changes in mitochondrial morphology and mitochondrial activity [32–39]. Thus, cellular oxidative metabolism was examined.

**Fig. 5:**
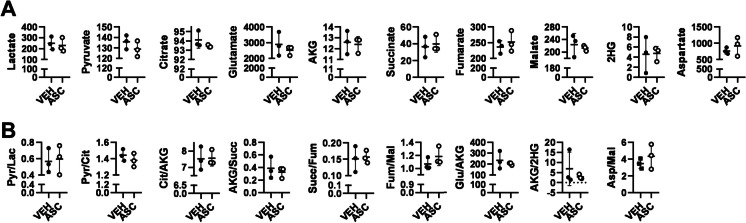
Ascites fluid treatment does not alter the steady-state levels of TCA cycle metabolites or anaplerotic metabolites of the TCA cycle. **A–B** A2780 cells (10^7^ cells/10 cm Petri-dish) were treated with 10% ascites fluid or PBS (vehicle). Each ascites sample was applied separately. Cells were harvested 2 h after treatment and subjected to steady-state metabolomics. All metabolites are expressed as ng/10^6^ cells except glutamate and aspartate, where absolute quantitation was not possible and the area under the curve normalized to cell numbers was used. The experiment was performed once using the three ascites samples. Control and ascites-treated samples were compared using *t*-tests. Abbreviations: AKG, α-ketoglutarate; ASC, ascites-treated; Asp, aspartate; Cit, citrate; Fum, fumarate; Glu, glutamate; Lac, lactate; Mal, malate; Pyr, pyruvate; Succ, succinate; Veh, vehicle; 2HG, 2-hydroxyglutarate

First, cellular metabolism was characterized using steady-state metabolomics centered around the tricarboxylic acid (TCA) cycle. No changes in the steady-state levels and ratios of metabolites of the TCA cycle and the anaplerotic metabolites to the TCA cycle were detected (Fig. 5A, B). In addition, no changes in the levels of 2-hydroxyglutarate, an oncometabolite with a prooncogenic role, were detected [40, 41].

**Fig. 6:**
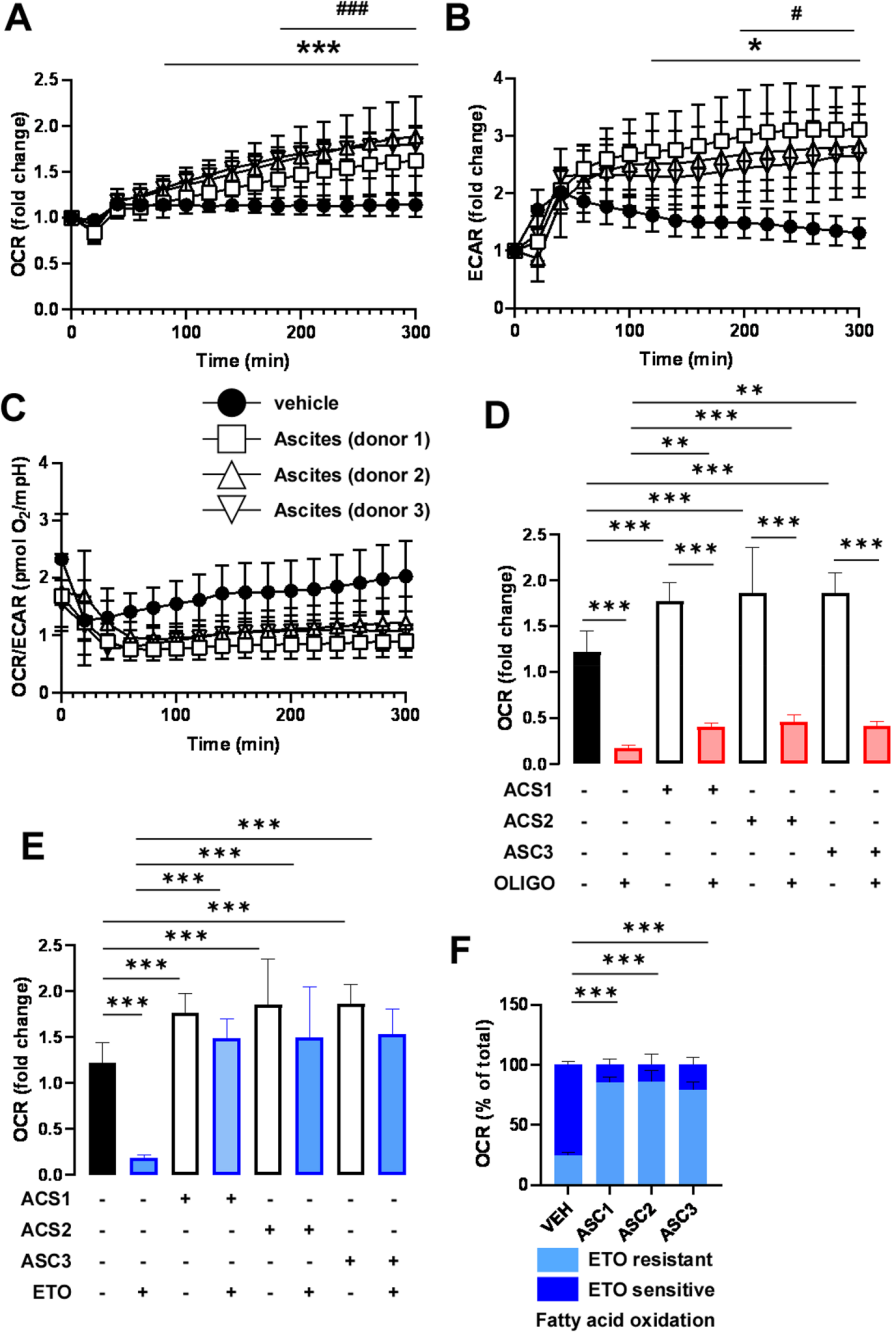
Treatment of A2780 cells with cell-free ascites induces Warburg-type metabolic rearrangement, induced uncoupling, and reduced fatty acid oxidation. **A–F** A2780 cells (20 000 cells/well) were plated in Seahorse assay plates and treated with 10% ascites (vol). **A** OCR and **B** ECAR values were recorded every 20 min. **C** The OCR/ECAR values are shown. After 5 h of treatment, cells were treated with oligomycin (2 µM) or etomoxir (50 µM) and OCR values were recorded. **D** Oligomycin-sensitive and oligomycin-resistant respiration was calculated. **E**–**F** Etomoxir-sensitive and etomoxir-resistant respiration was calculated. Experiments were repeated 3 times. Normality was assessed, and, if necessary, values were normalized using the Box-Cox normalization method. Statistical significance was assessed by a two-way ANOVA test followed by Dunnett’s post hoc test. In **A**–**C**, *, **, and *** represent differences between the zero time point and other time points at *p* < 0.05, *p* < 0.01, or *p* < 0.001, respectively. # and ### indicate statistical significance between vehicle-treated and ascites-treated cells at *p* < 0.05 or *p* < 0.001, respectively. In **D**–**F**, statistical significance was assessed by a two-way ANOVA test. * and *** indicate significant differences between the indicated groups at *p* < 0.05 or *p* < 0.001, respectively. Abbreviations: ASC, ascites-treated; ECAR, extracellular acidification rate; ETO, etomoxir; OCR, oxygen consumption rate; OLIGO, oligomycin

As no changes in steady-state metabolomics were detected in response to cell-free ascites, a Seahorse flux analysis was conducted, as this method enables the detection of changes to the flux of the pathways. Ascites treatment-induced mitochondrial oxidation as marked by enhanced oxygen consumption rate (OCR) (Fig. 6A) and extracellular acidification rate (ECAR), which is a proxy for glycolysis (Fig. 6B). Thus, cell-free ascites treatment rendered cells hypermetabolic. Furthermore, treatment of ovarian cancer cells with cell-free ascites decreased the OCR/ECAR ratio (Fig. 6C), which is a typical feature of Warburg-type rearrangement of metabolism [26].

To elucidate whether the oxidation of different substrates changes upon ascites treatment and assess uncoupled mitochondrial respiration, A2780 cells were treated with ascites for 5 h followed by treatment with inhibitors to block anaplerotic pathways. Fatty acid oxidation was blocked by etomoxir, glutamine oxidation by BPTES, and pyruvate oxidation by UK5099. To assess coupling, a complex V inhibitor oligomycin was applied. BPTES and UK5099 did not substantially influence substrate oxidation (data not shown); however, oligomycin treatment revealed that ascites treatment rendered increased mitochondrial uncoupling (Fig. 6D). Furthermore, ascites treatment decreased the level and proportion of fatty acid oxidation (Fig. 6E, F).

### Ascites-dependent induction of fatty acid oxidation depends on the TGFβ-ERK/MEK pathway

**Fig. 7:**
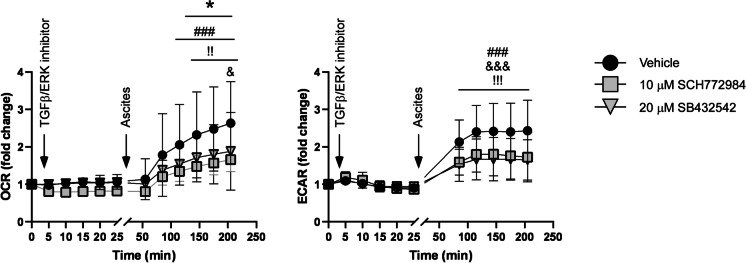
Pharmacological inhibition of TGFβ and ERK signaling attenuates ascites-induced metabolism. A2780 cells (20,000 cells/well) were plated in Seahorse assay plates. After recording baseline OCR and ECAR values, cells were treated with SB432542 or SCH772984 followed by the addition of ascites at the indicated time. Experiments were repeated 3 times. Ascites from patient 2 was used. Normality was assessed and, if necessary, values were normalized. Statistical significance was assessed by a one-way ANOVA test followed by Dunnett’s post hoc test. * indicates statistical significance between vehicle-treated vs. inhibitor-treated cells at *p* < 0.05. ### indicates significant differences between baseline and later time points in vehicle-treated cells at *p* < 0.001. &&& indicates significant differences between baseline and later time points in SCH772984-treated cells at *p* < 0.001. !! and !!! indicate significant differences between baseline and later time points in SB432542-treated cells at *p* < 0.01 or *p* < 0.001, respectively. Abbreviations: ECAR, extracellular acidification rate; OCR, oxygen consumption rate

The dependence of ascites-induced changes to core metabolic pathways on the activation of the TGFβ-ERK/MEK pathway was assessed using pharmacological inhibitors of the TGFβ type I receptor and ERK. SB432542 and SCH772984, which inhibit TGFβ type I receptor and ERK, did not affect OCR or ECAR but reduced ascites-induced increases in OCR and ECAR (Fig. 7). These results highlight the involvement of the TGFβ-ERK/MEK pathway in ascites-induced hypermetabolism in A2780 cells.

### The TGFβ signaling pathway affects disease outcome in cancers in humans

**Fig. 8:**
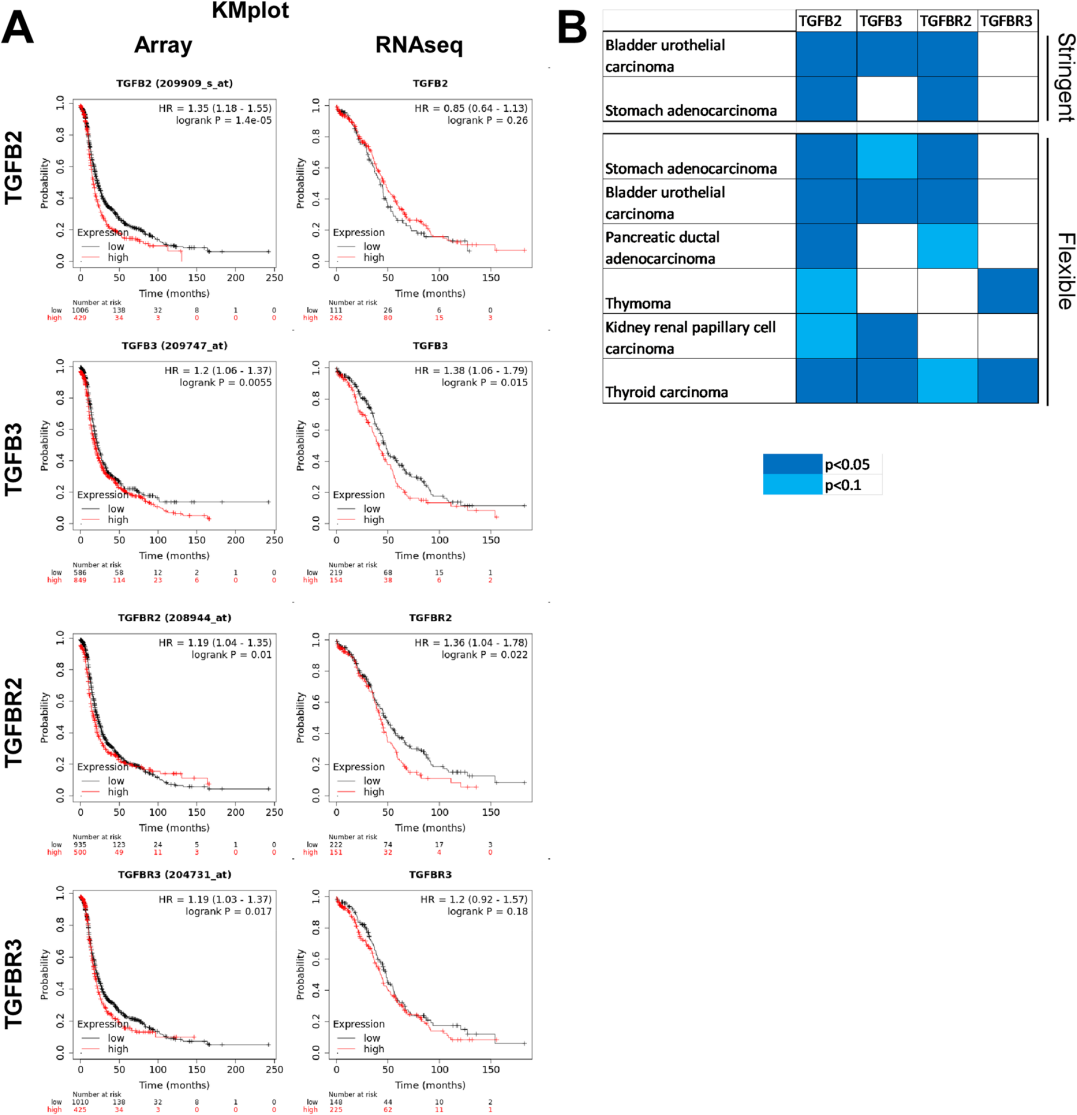
The overexpression of the members of TGFβ signaling pathway shorten survival in ovarian cancer and a subset of neoplasias. The effect of the expression of the isoforms of TGFβ and the TGFβ receptor on patient survival was assessed in **A** ovarian cancer and **B** in a subset of the indicated cancers. The kmplot.com database was accessed the 26th July 2023

As the above results implicated a role for the TGFβ signaling pathway in ovarian cancer progression. Therefore, we assessed how changes to the expression of TGFβ and TGFβ receptor (TGFBR) isoforms affect survival in ovarian cancer. First, we screened the effect of gene expression changes of TGFB1-3 and TGFBR1-3 on patient survival using the kmplot. com (both the RNAseq data and the microarray data) and the GEPIA2 database. TGFB1 and TGFBR1 expression did not affect survival (data not shown), while TGFB2, TGFB3, TGFBR2, and TGBR3 overexpression decreased patient survival among ovarian cancer patients when the microarray data was assessed (Fig. 8A). Similar changes were observed when the RNAseq dataset was probed of which the effect of TGBR3 was not significant, but had a similar trend as in the case of the microarray data (Fig. 8A). TGFB2 expression did not correlate with survival when the RNAseq data was assessed (Fig. 8A).

It remained an open question whether the TGFβ signaling pathway would be active in other cancers. To answer that question, we screened the pan-cancer database of the kmplot.com database and selected those neoplasias where at least one member of the overexpression of TGFB2 or TGB3 and TGFBR2 or TGBR3 negatively affected the overall survival. Data was evaluated in a stringent manner where *p* values less than 0.05 were considered and in a flexible way where *p* values less than 0.1 were considered, but at least one *p* value had be smaller than 0.05. The stringent evaluation bladder urothelial carcinoma and stomach adenocarcinoma was identified as neoplasias where TGFβ autocrine or paracrine signaling can feed forward carcinogenesis (Fig. 8B). Flexible evaluation revealed that TGFβ autocrine or paracrine signaling may feed forward pancreatic ductal adenocarcinoma, thymoma, kidney papillar cell carcinoma, and thyroid carcinoma (Fig. 8B). Of note, TGFβ autocrine or paracrine signaling can feed forward carcinogenesis only in a subset of neoplasias (3/21 in the case of stringent and 7/21 in the case of the flexible criteria).

## Discussion

**Fig. 9:**
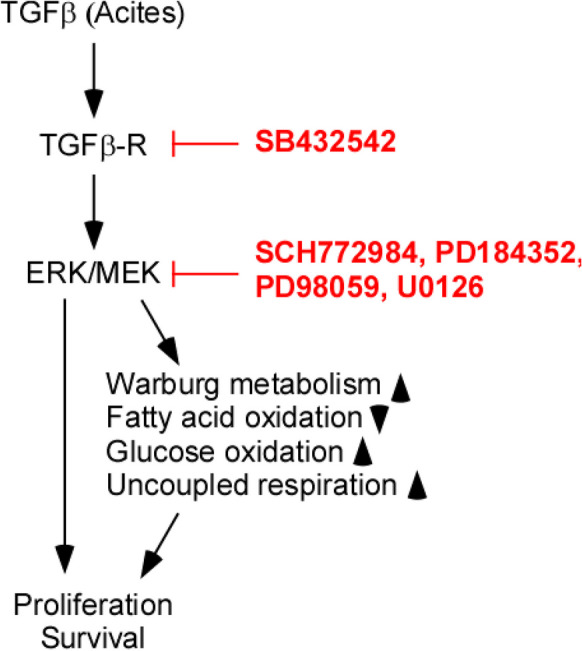
The overview of the ascitic TGFβ-elicited pathways. The applied pharmacological inhibitors are in red. Abbreviations: ERK, extracellular-signal-regulated kinases; MEK, mitogen-activated protein kinases; TGFβ, tumor growth factor β; TGFβ-R, tumor growth factor β receptor

The results of this study indicate that cell-free ascites from advanced-stage ovarian cancer patients induces cell proliferation in multiple models of ovarian cancer and untransformed, noncancerous primary human dermal fibroblasts. Previous studies showed that ascites has procarcinogenic effects associated with shear stress and increased intraabdominal pressure (reviewed in [8]). The flow of ascites can also transfer ovarian cancer cells within the abdominal cavity to support metastasis formation, and ascites promotes adhesion of ovarian cancer cells to peritoneal mesothelium and fibroblasts [42]. We extended these observations in our studies. The overview of the pathway identified in this study is on Fig. 9.

The complex composition of the cell-free fraction of ascites includes small molecular components and larger, peptide or protein-sized molecules. Although the components of ascites have been assessed in multiple studies [24, 43], the molecular composition of cell-free ascites is still poorly characterized. Using functional transcriptomics, we identified the TGFβ-induced signaling pathway as a key regulator of ovarian cancer cell proliferation. We demonstrated the presence of 0.96–2.94 ng/mL of TGFβ in cell-free ascites. TGFβ levels in ascites are comparable but in the lower half of the total TGFβ levels in the serum of healthy, unstimulated humans (range 1–7 ng/mL) [44–46]. A report by Santin et al. [46] demonstrated that serum TGFβ increases by roughly 50% in ovarian cancer patients compared to controls, highlighting the importance of TGFβ in ovarian cancer. Cell hyperproliferation was not limited to ovarian cancer cell lines, but occurred in untransformed human dermal fibroblasts also, suggesting a widespread effect among cells in contact with the ascites. Importantly, there is a parallel between the increased risk for ovarian cancer at older age [3–7] and the fact that low TGFβ is associated with successful aging [47, 48]. A TGFβ is an important component of the senescence-associated secretory phenotype (SASP) [49, 50], therefore, may be an important aging-associated secreted factor driving ovarian cancer progression. The cellular source of TGFβ in ascites is uncharacterized. It also remains an open question whether the ascites associated with ovarian cancer has similar composition and biological activity as the one associated with liver diseases.

Transcriptomics also suggested the involvement of MEKs and ERKs, which are cytoprotective and prooncogenic protein kinases [51–53] that play an active role in the progression of ovarian cancer [10, 54–56]. ERKs make widespread connections with other cytoprotective and prooncogenic pathways such as the Akt/mTOR pathway [55, 56]. Using pharmacological inhibitors of these pathways, we found that the TGFβ-ERK/MEK pathway elicits pro-proliferative effects on ovarian cancer cells, although the TGFβ-ERK/MEK pathway is likely not a single, exclusive, ascites-activated pathway.

Activation of the TGFβ-ERK pathway not only prompted cell proliferation but also induced Warburg metabolism in ovarian cancer cells. Importantly, the metabolic effects of cell-free ascites were drastic and uniform among patient samples. TGFβ-ERK/MEK activation is associated with mitochondrial morphology [33, 35, 36, 56], mitochondrial membrane potential [34], and mitochondrial permeability transition and cell death [32, 33]. Warburg rearrangement occurs in ovarian cancer cells [28–31], and changes to cellular metabolism are associated with metastatic capacity [8, 31, 37, 38, 57–61], suggesting that ascites-induced metabolic changes contribute to aggressive behavior in cancer cells. Ascites treatment induced hypermetabolism marked by increases in both OCR and ECAR values; nevertheless, as the OCR/ECAR ratio decreases, the hypermetabolic rearrangement of metabolism is dominated by the induction of glycolysis, which is a key feature of Warburg metabolism [26].

We observed changes to oligomycin-sensitive and etomoxir-sensitive respiration, highlighting changes to mitochondrial coupling and fatty acid oxidation in response to ascites. Increases in oligomycin-resistant respiration suggest a higher level of mitochondrial uncoupling. Oligomycin is an inhibitor of mitochondrial F1/F0 ATPase; therefore, the application of oligomycin reduces cellular oxygen consumption rate [62]. The rate of the oligomycin-resistant fraction of cellular respiration depends on mitochondrial uncoupling and correlates with the expression of uncoupling protein-1 [63]. Hence, increased oligomycin-resistant respiration suggests increases in uncoupled mitochondrial respiration. Mitochondrial uncoupling confers cell resistance against apoptosis in ovarian cancer (reviewed in [64]).

Ascites treatment induced the proportions and the levels of etomoxir-resistant respiration. Etomoxir is an inhibitor of carnitine-palmitoyltransferase (CPT) 1, which transfers acyl groups into the mitochondria for oxidation. Therefore, inhibition of CPT1 reduces fatty acid oxidation (termed etomoxir-sensitive respiration). In our study, the proportions of the etomoxir-sensitive respiration decreased in ascites-treated ovarian cancer cells, suggesting a decreased proportion of fatty acid degradation in cellular oxidative metabolism. Fatty acid metabolism is associated with ovarian cancer growth [65–71]. Importantly, a recent report [72] showed that the inhibition of CPT2 strongly supported metastasis formation and poor prognosis in a pool of human ovarian cancer patients. These results correlate with the ascites-induced reduction in fatty acid oxidation shown in this study.

## Conclusions and limitations

The results of this study indicate that cell-free ascites from ovarian cancer patients promotes the proliferation of ovarian cancer cell lines. Ascites treatment induced multiple pro-proliferative pathways in cultured ovarian cancer cells, most of which are associated with poor patient survival. including the TGFβ-ERK/MEK pathway. In addition, cell-free ascites induced a Warburg-type rearrangement of core metabolic pathways, including glycolysis and oxidative phosphorylation. Furthermore, cell-free ascites downregulated fatty acid oxidation and rendered the mitochondria less coupled. These pathways support tumor growth in a cooperative fashion. Importantly, the effects of ascites were uniform among different patients, suggesting that the procarcinogenic behavior is generalizable. Altogether, the soluble components of ascites seem to play a key role in supporting tumor progression in ovarian cancer; from a clinical perspective, these effects point towards the importance of reducing ascites production to reduce the risk of metastasis formation and cancer aggressiveness. Higher expression of the TGFβ signaling pathway is associated with shorter survival in ovarian cancer patients suggesting that the pathway we described hereby have implications in the human pathology. Furthermore, we identified a subset of human cancers where enhanced TGFβ signaling may correlate with shorter survival.

Our study has limitations that has to be considered for interpretation and when planning follow-up investigations. The number of patients involved (*n* = 3) is low despite the strong uniformity among the ascites samples. Along the same lines, the quantity of the ascites samples was limited that restricted the possible repeats of experiments. Finally, it remained an open question whether ascites associated with different pathologies (e.g., alcoholism) may support cell proliferation in the same manner.

## Materials and methods

### Chemicals

Routine chemicals were obtained from Sigma (St. Louis, MO, USA)) unless stated otherwise. Etomoxir (ETO) and oligomycin (OLIGO) were from Sigma (St. Louis, MO, USA). BPTES and UK5099 were from MedChemExpress (Monmouth Junction, NJ, USA). SB432542 was from Sigma and SCH772984 was from Selleckchem (Houston, TX, USA).

### Ascites donors

Ascites was collected from advanced-stage ovarian cancer patients undergoing paracentesis. The local ethical board authorized the study under the number RKEB.5829. Patients signed an informed consent declaration. Characteristics of the patients are shown in Table 1. None of the patients showed clinical signs of platinum resistance. All ascites samples tested negative for lipopolysaccharide content (GyEL LTD., Budapest, Hungary; turbidimetric LAL assay technique).

**Table 1:** Clinical data on the ascites donor patients

Patient	Diagnosis	Age	TNM stage	FIGO
1	High-grade serous carcinoma	65	T3 N0 M1	IV
2	Serous carcinoma	77	T3 N0 M1	IV
3	Serous carcinoma	80	T3 N0 M0	III/C

### Cell culture

A2780 cells were cultured in RMPI 1640 medium supplemented with 10% fetal calf serum, 2 mM glutamine, and 1% penicillin–streptomycin. ID8 cells were cultured in high glucose DMEM (4.5 g/L glucose) medium supplemented with 4% fetal calf serum, 2 mM glutamine, 1% penicillin–streptomycin, and 1% ITS supplement (I3146). Human primary dermal fibroblasts were cultured in low glucose DMEM (1 g/L glucose) medium supplemented with 20% fetal calf serum, 2 mM glutamine, and 1% penicillin–streptomycin. All cells were confirmed mycoplasma free by PCR analysis and were regularly tested during the experiments. Both cell lines were used in passage numbers lower than 30.

### Determination of TGFβ in ascites

TGFβ was measured in ascites samples using an ELISA kit from Invitrogen (Waltham, MA, USA, cat. no. BMS249-4). Measurements were performed according to the manufacturer’s instructions. The “sigmoidal, 4PL” formula was used to fit the standard curve.

### Sulforhodamine B (SRB) assay

Proliferation was assessed using the SRB assay [73–75]. Cells were seeded in 96 well plates the day before the assay (A2780: 1000 cells/well, ID8 cells: 1000 cells/well, primary human dermal fibroblasts: 3000 cells/well). Cells were treated with the ascites in 10% of the volume (180 µL medium + 20 µL ascites or PBS as vehicle) for 48 h. Then medium was removed and cells were fixed with 10% trichloroacetic acid. Fixed cells were washed in distilled water 3 times followed by staining with SRB (0.4 m/V% dissolved in 1% acetic acid) for 10 min. Stained cells were washed in 1% acetic acid 5 times; acetic acid was removed and cells were left to dry. Protein-bound SRB was released by adding 100 µl 10 mM Tris base. Plates were measured in a plate photometer (Thermo Scientific Multiscan GO spectrophotometer, Waltham, MA, USA) at 540 nm. On each plate wells were designed to contain vehicle-treated cells. In calculations, the readings for these wells were considered as 1, and all readings were expressed relative to these values.

### Oximetry

Seahorse indirect oximetry was performed to determine cellular oxygen consumption (OCR) and extracellular acidification (ECAR) applying the considerations described in [76, 77]. Briefly, A2780 cells were seeded into Seahorse XF96 Cell Culture Microplates at a concentration of 20,000 cells/well in 200 µl growth media. Cells were incubated at 37°C and 5% CO_2_ overnight. On the following day, the growth media was changed to assay media (XF Assay Media Modified DMEM with 2 g/L glucose). Desired compounds were loaded into an appropriate port of the cartridge (20 µl into port A, 22 µl into port B, 24 µl into port C, and 26 µl into port D). Data integrations were performed for 5 min for all measurement points.

To assess the effects of ascites treatment on A2780 cells, 5 measurements were performed without any treatment to record the baseline oxygen consumption rate. Then, 10% ascites (vol/vol) was added, and data was recorded every 20 min for 300 min. Etomoxir (ETO) was added and 5 recordings were performed. Subsequently, oligomycin (OLIGO) was added and 5 recordings were performed. Finally, to measure background OCR, antimycin was added and OCR was recorded 5 times.

To verify the link between ascites treatment and TGFβ-ERK signaling, we used a different protocol. First, 5 measurements were performed without any treatment to record the baseline oxygen consumption rate. Next, SB432542, SCH772984, or vehicle was added to cells, and 5 measurements were performed. Then, 10% ascites was added to wells, and data was recorded every 30 min for 240 min. Finally, to measure background OCR, antimycin was added, and OCR was recorded 5 times.

Antimycin readings were subtracted from OCR reads for each well. To assess changes to EACR and OCR, fold changes were calculated for each well. To assess the OCR/ECAR value, the actual nominal ECAR and OCR-antimycin values were used.

### Liquid chromatography–mass spectrometry

Intracellular metabolite (citrate, malate, pyruvate, lactate, succinate, fumarate, glutamate, aspartate, α-ketoglutarate) analysis was performed, as described previously [78]. In brief, 1 × 10^6^ cells were quenched in liquid nitrogen, and metabolites were extracted from cells with methanol–chloroform–water. After centrifugation (15,000 × g, 10 min, 4 °C), the supernatants were stored at − 80 °C until the measurements. The range of metabolite concentrations was assessed using calibration curves. The dilution of the analytical grade standards was 0.5–50 µM. Concentrations are expressed as ng/10^6^ cells, except amino acids, where only AUC was calculated. Liquid chromatography–mass spectrometry (LC–MS) analysis was performed on an Agilent 1100 Series high-performance LC system coupled with a Sciex QTRAP 6500 mass spectrometer. Chromatographic separation was performed on a Halo Penta-HILIC column (150 × 2.1 mm, 2.7 µm) (Hichrom Ltd., Berkshire, UK). Two mobile phases were used (A: 10 mM ammonium formate at pH = 5; B: acetonitrile containing 0.1% (v/v) formic acid), and the mass spectrometry was run in negative electrospray ionization mode. For the measurements, the following setup was adjusted: source temperature, 450 °C; ionization voltage, − 4500 V; entrance potential, − 10 V; curtain gas, 45 psi; gas 1, 45 psi; gas 2, 35 psi; CAD gas, medium. For LC–MS analyses, two independent experimental measurements with a minimum of three parallel samples were performed.

### RNA sequencing

To obtain global transcriptome data, high-throughput mRNA sequencing analysis was performed on the Illumina sequencing platform. Total RNA was isolated using the Eukaryotic Total RNA Nano Kit according to the manufacturer’s protocol quality and checked on an Agilent BioAnalyzer. Samples with RNA integrity number value > 7 were accepted for library preparation.

RNA-Seq libraries were prepared from total RNA using an Ultra II RNA Sample Prep kit (New England BioLabs) according to the manufacturer’s protocol. Briefly, poly-A RNAs were captured by oligo-dT conjugated magnetic beads, and the mRNAs were eluted and fragmented at 94°C. First-strand cDNA was generated by random priming reverse transcription, and after the second-strand synthesis step, double-stranded cDNA was generated. After repairing ends, A-tailing, and the adapter ligation steps, adapter-ligated fragments were amplified in enrichment PCR, and finally sequencing libraries were generated. Sequencing runs were executed on an Illumina NextSeq 500 instrument using single-end 75-cycle sequencing.

### RNA sequencing data analysis

Raw sequencing data (fastq) was aligned to the human reference genome version GRCh38 using a HISAT2 algorithm and BAM files were generated. Downstream analysis was performed using StrandNGS software (www.strand-ngs.com). BAM files were imported into the software DESeq algorithm for normalization. A moderated *t*-test was used to determine differentially expressed genes between conditions.

### Pathway analyses

CytoScape v3.4 software with the ClueGo v2.3.5. application was used for identifying over-represented gene ontology (GO) terms. A two-sided hypergeometric test was performed, and the list of differentially expressed genes was tested against GO biological process, Reactome, and KEGG Pathway databases.

### Database screening

The kmplot.com database [25] was used to assess the relationship between tumoral gene expression and patient survival. The tissue distribution of the TGFβ signaling pathway was assessed in the GTEx portal [79]. Aging-associated changes to the TGFβ signaling pathway was assessed using the GenAge database [80]. The literature resources provided by the GenAge database were reviewed by the authors, and the relevant publications were incorporated into the manuscript.

### Statistical analyses

All experiments were repeated at least 3 three times. All graphs and statistical analyses were generated using GraphPad Prism v.8.0.1 software. Normal distribution of the data was assessed using the Shapiro–Wilk test. Statistical tests are indicated in the figure legends, and the statistical tests can be accessed in the primary data files. Box-Cox transformation was performed as described in [81].

### Ethics approval and consent to participate

Ascites was collected from advanced-stage ovarian cancer patients undergoing paracentesis. The local ethical board authorized the study under the number RKEB.5829. Patients signed an informed consent declaration.

### Supplementary Information

Below is the link to the electronic supplementary material.

Supplementary file1 (DOCX 182 KB)

Supplementary file2 (XLSX 11 KB)

## Declarations

### Data availability

Primary data is available at the Figshare.com repository https://figshare.com/s/f530fc96698059ec4f8610.6084/m9.figshare.21543162). The results of the RNA sequencing dataset were uploaded to the NCBI SRA database (Submission ID: SUB12949677; BioProject ID: PRJNA944542).

## Abbreviations

AKG: α-Ketoglutarate
ASC: Ascites-treated/ascites
Asp: Aspartate
Cit: Citrate
CPT: Carnitine-palmitoyltransferase
ECAR: Extracellular acidification
ERK: Extracellular-signal-regulated kinase
ETO: Etomoxir
Fum: Fumarate
Glu: Glutamate
GO: Gene ontology
Lac: Lactate
Mal: Malate
NTRK: Neurotrophic tyrosine receptor kinase
OCR: Oxygen consumption rate
OLIGO: Oligomycin
Pyr: Pyruvate
SRB: Sulforhodamine B
Succ: Succinate
TCA: Tricarboxylic acid (cycle)
TGF: Tumor growth factor
2HG: 2-Hydroxyglutarate
