# Supplementary material for: Cell-free ascites from ovarian cancer patients induces Warburg metabolism and cell proliferation through TGFβ-ERK signaling
Source: GeroScience. 2024 Jan 10;46(4):3581–97. doi: 10.1007/s11357-023-01056-1 (PMC11226691; doi:10.1007/s11357-023-01056-1)
Supplement: Supplementary file 1 — Supplementary file1 (DOCX 182 KB) [file 11357_2023_1056_MOESM1_ESM.docx]

**Supplementary materials for Szeőcs et al. “Cell-free ascites from ovarian cancer patients induces Warburg metabolism and cell proliferation through TGFβ-ERK signaling”**

**Supplementary Figures**

**Supplementary Figure 1. The depiction of the results of the biological process reactome pathway analysis**


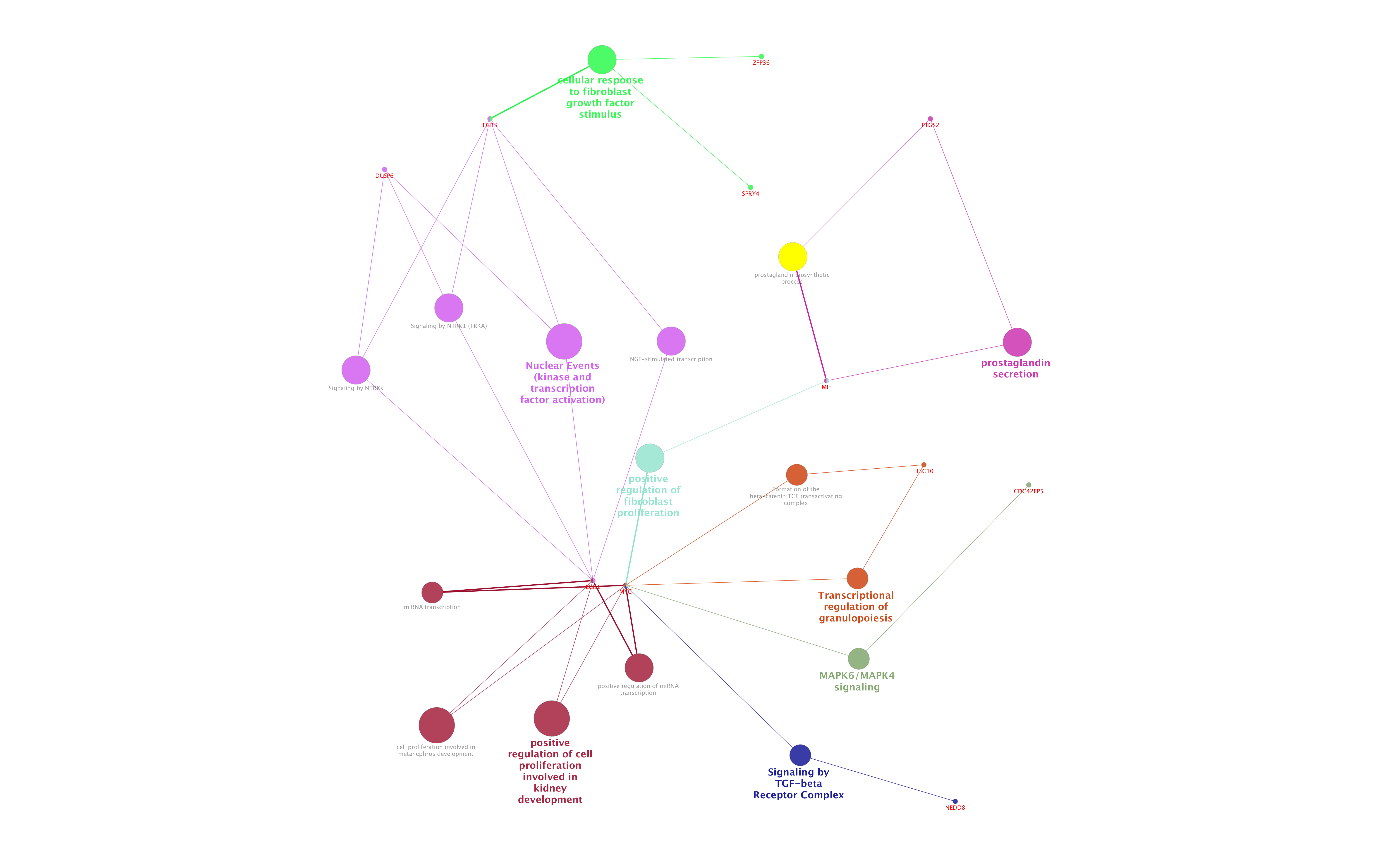


**Supplementary Figure 2. The depiction of the results of the KEGG pathway analysis**


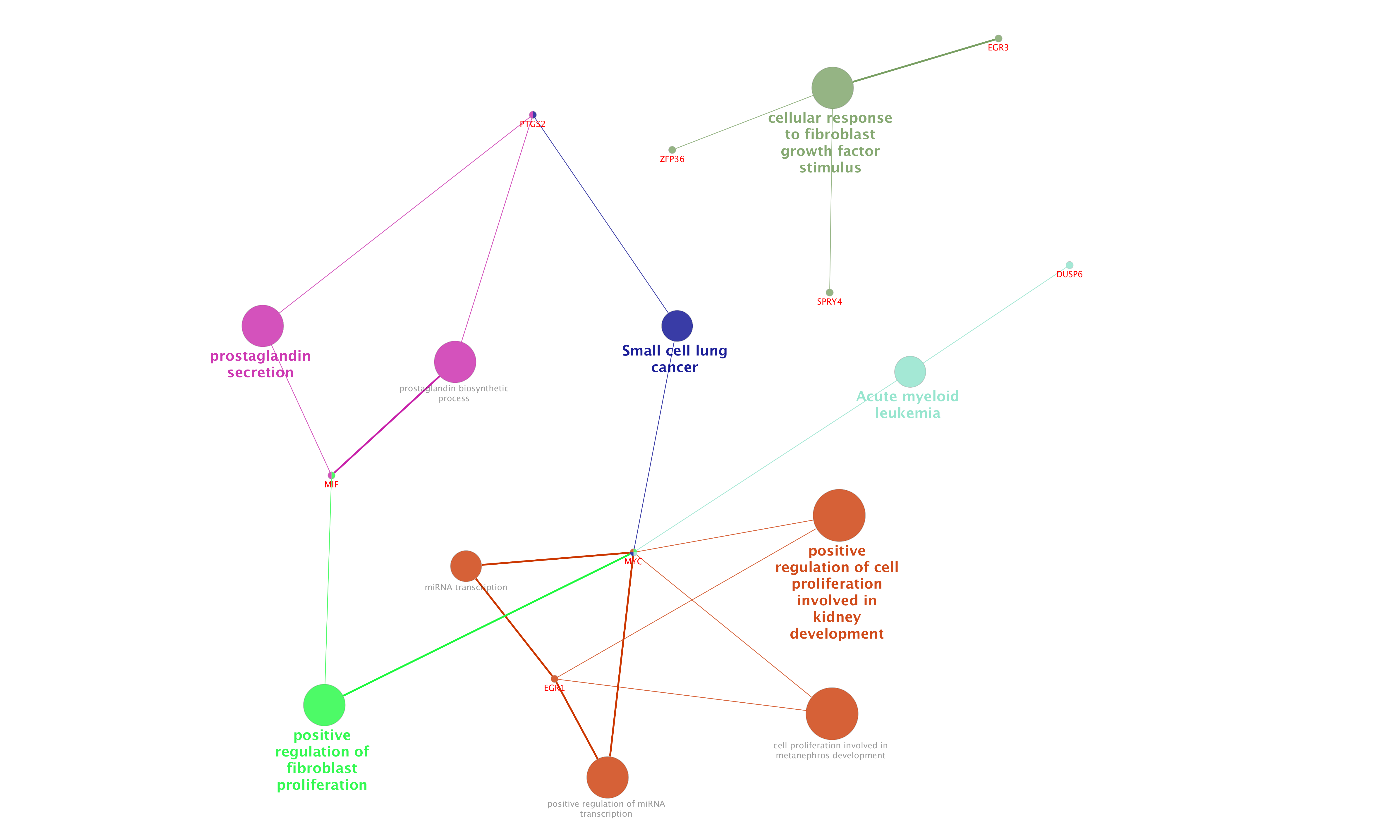


**Supplelmentary Table 1. The list of the genes with dysregulated mRNA expression upon ascites treatment in A2780 cells**

| **Regulation** | **True FC** | **Gene ID** | **p** | **Gene Symbol** | **Description** |
| --- | --- | --- | --- | --- | --- |
| up | 2,9477072 | ENSG00000200237 | 3.6726982E-4 | SNORA70 | Small nucleolar RNA SNORA70 [Source:RFAM;Acc:RF00156] |
| up | 2,7018178 | ENSG00000229780 | 0.0016217054 | UBE2Q1-AS1 | UBE2Q1 antisense RNA 1 [Source:HGNC Symbol;Acc:HGNC:40722] |
| up | 2,5710666 | ENSG00000120738 | 0.0025615287 | EGR1 | early growth response 1 [Source:HGNC Symbol;Acc:HGNC:3238] |
| up | 2,192126 | ENSG00000264425 | 6.1804353E-6 | MIR4653 | microRNA 4653 [Source:HGNC Symbol;Acc:HGNC:41562] |
| up | 2,179043 | ENSG00000266563 | 0.0076324726 | EIF1P5 | eukaryotic translation initiation factor 1 pseudogene 5 [Source:HGNC Symbol;Acc:HGNC:49618] |
| up | 2,1386595 | ENSG00000179388 | 3.9478986E-5 | EGR3 | early growth response 3 [Source:HGNC Symbol;Acc:HGNC:3240] |
| up | 1,9871191 | ENSG00000230165 | 0.01687274 | AURKAPS2 | aurora kinase A pseudogene 2 [Source:HGNC Symbol;Acc:HGNC:18612] |
| up | 1,9435551 | ENSG00000232797 | 0.023352714 | FAM207CP | family with sequence similarity 207 member C, pseudogene [Source:HGNC Symbol;Acc:HGNC:42676] |
| up | 1,7827168 | ENSG00000207187 | 0.02124332 | SNORA64 | Small nucleolar RNA SNORA64/SNORA10 family [Source:RFAM;Acc:RF00264] |
| up | 1,7094568 | ENSG00000073756 | 5.1847426E-4 | PTGS2 | prostaglandin-endoperoxide synthase 2 [Source:HGNC Symbol;Acc:HGNC:9605] |
| up | 1,6764557 | ENSG00000264585 | 0.041079063 | MIR4449 | microRNA 4449 [Source:HGNC Symbol;Acc:HGNC:41864] |
| up | 1,6551335 | ENSG00000237786 | 2.2137059E-4 | GFOD1-AS1 | GFOD1 antisense RNA 1 [Source:HGNC Symbol;Acc:HGNC:40956] |
| up | 1,6327174 | ENSG00000221420 | 6.6429275E-5 | SNORA81 | small nucleolar RNA, H/ACA box 81 [Source:HGNC Symbol;Acc:HGNC:32667] |
| up | 1,6224425 | ENSG00000271550 | 0.0029100254 | BNIP3P11 | BCL2 interacting protein 3 pseudogene 11 [Source:HGNC Symbol;Acc:HGNC:49724] |
| up | 1,6073337 | ENSG00000167617 | 0.0022706757 | CDC42EP5 | CDC42 effector protein 5 [Source:HGNC Symbol;Acc:HGNC:17408] |
| up | 1,5232219 | ENSG00000267541 | 0.017208245 | MTCO2P2 | mitochondrially encoded cytochrome c oxidase II pseudogene 2 [Source:HGNC Symbol;Acc:HGNC:25354] |
| up | 1,5137937 | ENSG00000265806 | 0.03795629 | MIR4292 | microRNA 4292 [Source:HGNC Symbol;Acc:HGNC:38348] |
| up | 1,5077088 | ENSG00000231940 | 0.04937398 | RPS7P3 | ribosomal protein S7 pseudogene 3 [Source:HGNC Symbol;Acc:HGNC:36518] |
| up | 1,4689386 | ENSG00000183055 | 0.011377843 | FAM133CP | family with sequence similarity 133 member C, pseudogene [Source:HGNC Symbol;Acc:HGNC:44190] |
| up | 1,4523611 | ENSG00000277985 | 0.0076470156 | SNORA67 | small nucleolar RNA, H/ACA box 67 [Source:HGNC Symbol;Acc:HGNC:10224] |
| up | 1,4276246 | ENSG00000275559 | 0.014187338 | DLEU2_6 | Deleted in lymphocytic leukemia 2 conserved region 6 [Source:RFAM;Acc:RF02110] |
| up | 1,4275063 | ENSG00000214832 | 0.032082353 | UPF3AP2 | UPF3A pseudogene 2 [Source:HGNC Symbol;Acc:HGNC:30567] |
| up | 1,4143887 | ENSG00000164845 | 0.0032925047 | FAM86FP | family with sequence similarity 86 member F, pseudogene [Source:HGNC Symbol;Acc:HGNC:42357] |
| up | 1,4070482 | ENSG00000228288 | 0.001366682 | PCAT6 | prostate cancer associated transcript 6 (non-protein coding) [Source:HGNC Symbol;Acc:HGNC:43714] |
| up | 1,4037645 | ENSG00000128016 | 0.02235977 | ZFP36 | ZFP36 ring finger protein [Source:HGNC Symbol;Acc:HGNC:12862] |
| up | 1,3625283 | ENSG00000104044 | 0.011527806 | OCA2 | OCA2 melanosomal transmembrane protein [Source:HGNC Symbol;Acc:HGNC:8101] |
| up | 1,3567642 | ENSG00000225263 | 0.023512835 | PCDH9-AS3 | PCDH9 antisense RNA 3 [Source:HGNC Symbol;Acc:HGNC:40427] |
| up | 1,3556095 | ENSG00000223768 | 0.005666636 | LINC00205 | long intergenic non-protein coding RNA 205 [Source:HGNC Symbol;Acc:HGNC:16420] |
| up | 1,3456439 | ENSG00000213977 | 0.04334673 | TAX1BP3 | Tax1 binding protein 3 [Source:HGNC Symbol;Acc:HGNC:30684] |
| up | 1,3336275 | ENSG00000115641 | 0.003807424 | FHL2 | four and a half LIM domains 2 [Source:HGNC Symbol;Acc:HGNC:3703] |
| up | 1,3330634 | ENSG00000205583 | 0.048959848 | STAG3L1 | stromal antigen 3-like 1 (pseudogene) [Source:HGNC Symbol;Acc:HGNC:33852] |
| up | 1,3328687 | ENSG00000224953 | 0.03022431 | SRIP3 | sorcin pseudogene 3 [Source:HGNC Symbol;Acc:HGNC:38735] |
| up | 1,3266606 | ENSG00000147804 | 0.016489785 | SLC39A4 | solute carrier family 39 member 4 [Source:HGNC Symbol;Acc:HGNC:17129] |
| up | 1,3235443 | ENSG00000233111 | 0.046089772 | RAB1C | RAB1C, member RAS oncogene family pseudogene [Source:HGNC Symbol;Acc:HGNC:23683] |
| up | 1,3233577 | ENSG00000139318 | 0.0044674734 | DUSP6 | dual specificity phosphatase 6 [Source:HGNC Symbol;Acc:HGNC:3072] |
| up | 1,3196259 | ENSG00000142871 | 0.00361219 | CYR61 | cysteine rich angiogenic inducer 61 [Source:HGNC Symbol;Acc:HGNC:2654] |
| up | 1,3176638 | ENSG00000236044 | 0.0014407363 | FABP5P2 | fatty acid binding protein 5 pseudogene 2 [Source:HGNC Symbol;Acc:HGNC:31060] |
| up | 1,3144366 | ENSG00000187678 | 0.03410386 | SPRY4 | sprouty RTK signaling antagonist 4 [Source:HGNC Symbol;Acc:HGNC:15533] |
| up | 1,3125435 | ENSG00000276314 | 0.025724834 | SNORD107 | small nucleolar RNA, C/D box 107 [Source:HGNC Symbol;Acc:HGNC:32771] |
| up | 1,3071051 | ENSG00000237883 | 0.039194833 | DGUOK-AS1 | DGUOK antisense RNA 1 [Source:HGNC Symbol;Acc:HGNC:43441] |
| up | 1,2937657 | ENSG00000104447 | 0.016541978 | TRPS1 | transcriptional repressor GATA binding 1 [Source:HGNC Symbol;Acc:HGNC:12340] |
| up | 1,2896274 | ENSG00000231468 | 0.032214776 | PRDX3P2 | peroxiredoxin 3 pseudogene 2 [Source:HGNC Symbol;Acc:HGNC:39265] |
| up | 1,2879456 | ENSG00000231707 | 0.035567768 | PABPC1P1 | poly(A) binding protein cytoplasmic 1 pseudogene 1 [Source:HGNC Symbol;Acc:HGNC:8558] |
| up | 1,2853307 | ENSG00000278828 | 0.019756837 | HIST1H3H | histone cluster 1 H3 family member h [Source:HGNC Symbol;Acc:HGNC:4775] |
| up | 1,2839777 | ENSG00000136997 | 0.014050976 | MYC | v-myc avian myelocytomatosis viral oncogene homolog [Source:HGNC Symbol;Acc:HGNC:7553] |
| up | 1,267008 | ENSG00000215784 | 0.021528244 | FAM72D | family with sequence similarity 72 member D [Source:HGNC Symbol;Acc:HGNC:33593] |
| up | 1,2619482 | ENSG00000159840 | 0.030144261 | ZYX | zyxin [Source:HGNC Symbol;Acc:HGNC:13200] |
| up | 1,2524849 | ENSG00000218996 | 0.023981577 | ARL4AP5 | ADP ribosylation factor like GTPase 4A pseudogene 5 [Source:HGNC Symbol;Acc:HGNC:52374] |
| up | 1,2479565 | ENSG00000155090 | 0.035589546 | KLF10 | Kruppel like factor 10 [Source:HGNC Symbol;Acc:HGNC:11810] |
| up | 1,2470483 | ENSG00000181227 | 0.034849685 | DLSTP1 | dihydrolipoamide S-succinyltransferase pseudogene 1 [Source:HGNC Symbol;Acc:HGNC:2912] |
| down | -1,2514668 | ENSG00000119938 | 0.04149522 | PPP1R3C | protein phosphatase 1 regulatory subunit 3C [Source:HGNC Symbol;Acc:HGNC:9293] |
| down | -1,266786 | ENSG00000283277 | 0.045701448 | FBXO31 | F-box protein 31 [Source:HGNC Symbol;Acc:HGNC:16510] |
| down | -1,2669684 | ENSG00000240204 | 0.010574033 | SMKR1 | small lysine rich protein 1 [Source:HGNC Symbol;Acc:HGNC:43561] |
| down | -1,2720221 | ENSG00000253327 | 0.010656143 | RAD21-AS1 | RAD21 antisense RNA 1 [Source:HGNC Symbol;Acc:HGNC:32158] |
| down | -1,2891599 | ENSG00000221883 | 0.027453175 | ARIH2OS | ariadne homolog 2 opposite strand [Source:HGNC Symbol;Acc:HGNC:34425] |
| down | -1,2986972 | ENSG00000236152 | 0.017886551 | MRPS36P1 | mitochondrial ribosomal protein S36 pseudogene 1 [Source:HGNC Symbol;Acc:HGNC:29771] |
| down | -1,3027892 | ENSG00000271672 | 0.047135614 | DUXAP8 | double homeobox A pseudogene 8 [Source:HGNC Symbol;Acc:HGNC:32187] |
| down | -1,3240199 | ENSG00000277072 | 0.027823128 | STAG3L2 | stromal antigen 3-like 2 (pseudogene) [Source:HGNC Symbol;Acc:HGNC:33886] |
| down | -1,3281302 | ENSG00000267691 | 0.010456534 | SHC1P2 | SHC adaptor protein 1 pseudogene 2 [Source:HGNC Symbol;Acc:HGNC:10842] |
| down | -1,3439653 | ENSG00000276168 | 0.022338718 | RN7SL1 | RNA, 7SL, cytoplasmic 1 [Source:HGNC Symbol;Acc:HGNC:10038] |
| down | -1,3547242 | ENSG00000254835 | 0.04004662 | RNF185-AS1 | RNF185 antisense RNA 1 [Source:HGNC Symbol;Acc:HGNC:41161] |
| down | -1,3561497 | ENSG00000226981 | 0.02642097 | ABHD17AP6 | abhydrolase domain containing 17A pseudogene 6 [Source:HGNC Symbol;Acc:HGNC:34044] |
| down | -1,378499 | ENSG00000257446 | 0.0017799074 | ZNF878 | zinc finger protein 878 [Source:HGNC Symbol;Acc:HGNC:37246] |
| down | -1,3803091 | ENSG00000236679 | 0.041618254 | RPL23AP24 | ribosomal protein L23a pseudogene 24 [Source:HGNC Symbol;Acc:HGNC:36176] |
| down | -1,3877183 | ENSG00000240972 | 0.0033792865 | MIF | macrophage migration inhibitory factor (glycosylation-inhibiting factor) [Source:HGNC Symbol;Acc:HGNC:7097] |
| down | -1,4054805 | ENSG00000263740 | 6.9457164E-6 | RN7SL4P | RNA, 7SL, cytoplasmic 4, pseudogene [Source:HGNC Symbol;Acc:HGNC:10039] |
| down | -1,4119443 | ENSG00000129559 | 0.0014465789 | NEDD8 | neural precursor cell expressed, developmentally down-regulated 8 [Source:HGNC Symbol;Acc:HGNC:7732] |
| down | -1,4263561 | ENSG00000274012 | 0.0010322204 | RN7SL2 | RNA, 7SL, cytoplasmic 2 [Source:HGNC Symbol;Acc:HGNC:23134] |
| down | -1,4359555 | ENSG00000225423 | 0.009289591 | TNPO1P1 | transportin 1 pseudogene 1 [Source:HGNC Symbol;Acc:HGNC:45120] |
| down | -1,4372994 | ENSG00000231789 | 1.5836447E-6 | PIK3CD-AS2 | PIK3CD antisense RNA 2 [Source:HGNC Symbol;Acc:HGNC:51334] |
| down | -1,4405223 | ENSG00000172058 | 0.037489835 | SERF1A | small EDRK-rich factor 1A [Source:HGNC Symbol;Acc:HGNC:10755] |
| down | -1,4682686 | ENSG00000104894 | 0.0155963795 | CD37 | CD37 molecule [Source:HGNC Symbol;Acc:HGNC:1666] |
| down | -1,4721034 | ENSG00000148290 | 0.01686941 | SURF1 | SURF1, cytochrome c oxidase assembly factor [Source:HGNC Symbol;Acc:HGNC:11474] |
| down | -1,583577 | ENSG00000250412 | 0.03880814 | KLHL2P1 | kelch like family member 2 pseudogene 1 [Source:HGNC Symbol;Acc:HGNC:44046] |
| down | -1,612305 | ENSG00000231500 | 0.02033639 | RPS18 | ribosomal protein S18 [Source:HGNC Symbol;Acc:HGNC:10401] |
| down | -1,6299042 | ENSG00000200913 | 0.04580613 | SNORD46 | small nucleolar RNA, C/D box 46 [Source:HGNC Symbol;Acc:HGNC:10186] |
| down | -1,6430213 | ENSG00000278771 | 0.009891675 | Metazoa_SRP | Metazoan signal recognition particle RNA [Source:RFAM;Acc:RF00017] |
| down | -1,6745209 | ENSG00000231240 | 0.02600546 | KLF2P1 | Kruppel like factor 2 pseudogene 1 [Source:HGNC Symbol;Acc:HGNC:49280] |
| down | -1,7078915 | ENSG00000259419 | 0.009440136 | HNRNPCP3 | heterogeneous nuclear ribonucleoprotein C pseudogene 3 [Source:HGNC Symbol;Acc:HGNC:48815] |
| down | -1,889383 | ENSG00000221184 | 0.0024979 | MIR1254-1 | microRNA 1254-1 [Source:HGNC Symbol;Acc:HGNC:35319] |
| down | -1,9506652 | ENSG00000205544 | 0.036315683 | TMEM256 | transmembrane protein 256 [Source:HGNC Symbol;Acc:HGNC:28618] |
| down | -2,0208325 | ENSG00000255526 | 9.0364943E-4 | NEDD8-MDP1 | NEDD8-MDP1 readthrough [Source:HGNC Symbol;Acc:HGNC:39551] |
| down | -2,6719124 | ENSG00000212385 | 0.004469624 | RNU6-817P | RNA, U6 small nuclear 817, pseudogene [Source:HGNC Symbol;Acc:HGNC:47780] |
| down | -3,5985348 | ENSG00000278039 | 0.043509685 | Xist_exon1 | X-chromosome inactivation gene exon 1 [Source:RFAM;Acc:RF01880] |

**Supplementary Table 2. The results of the biological process reactome pathway analysis**

Non corrected p values are indicated in the table.

| ID | Term | Ontology Source | p value | Associated Genes |
| --- | --- | --- | --- | --- |
| R-HSA:5687128 | MAPK6/MAPK4 signaling | REACTOME_Pathways_25.05.2022 | 0,0087044 | [CDC42EP5, MYC] |
| GO:0044344 | cellular response to fibroblast growth factor stimulus | GO_BiologicalProcess-EBI-UniProt-GOA-ACAP-ARAP_25.05.2022_00h00 | 0,0007243 | [EGR3, SPRY4, ZFP36] |
| GO:0048146 | positive regulation of fibroblast proliferation | GO_BiologicalProcess-EBI-UniProt-GOA-ACAP-ARAP_25.05.2022_00h00 | 0,0039164 | [MIF, MYC] |
| R-HSA:170834 | Signaling by TGF-beta Receptor Complex | REACTOME_Pathways_25.05.2022 | 0,0087044 | [MYC, NEDD8] |
| GO:0032310 | prostaglandin secretion | GO_BiologicalProcess-EBI-UniProt-GOA-ACAP-ARAP_25.05.2022_00h00 | 0,0005483 | [MIF, PTGS2] |
| GO:0001516 | prostaglandin biosynthetic process | GO_BiologicalProcess-EBI-UniProt-GOA-ACAP-ARAP_25.05.2022_00h00 | 0,0013943 | [MIF, PTGS2] |
| R-HSA:201722 | Formation of the beta-catenin:TCF transactivating complex | REACTOME_Pathways_25.05.2022 | 0,0090846 | [H3C10, MYC] |
| R-HSA:9616222 | Transcriptional regulation of granulopoiesis | REACTOME_Pathways_25.05.2022 | 0,0088936 | [H3C10, MYC] |
| R-HSA:166520 | Signaling by NTRKs | REACTOME_Pathways_25.05.2022 | 0,0012172 | [DUSP6, EGR1, EGR3] |
| R-HSA:187037 | Signaling by NTRK1 (TRKA) | REACTOME_Pathways_25.05.2022 | 0,0007821 | [DUSP6, EGR1, EGR3] |
| R-HSA:198725 | Nuclear Events (kinase and transcription factor activation) | REACTOME_Pathways_25.05.2022 | 0,0001207 | [DUSP6, EGR1, EGR3] |
| R-HSA:9031628 | NGF-stimulated transcription | REACTOME_Pathways_25.05.2022 | 0,0017297 | [EGR1, EGR3] |
| GO:0072203 | cell proliferation involved in metanephros development | GO_BiologicalProcess-EBI-UniProt-GOA-ACAP-ARAP_25.05.2022_00h00 | 0,0001320 | [EGR1, MYC] |
| GO:1901724 | positive regulation of cell proliferation involved in kidney development | GO_BiologicalProcess-EBI-UniProt-GOA-ACAP-ARAP_25.05.2022_00h00 | 0,0000865 | [EGR1, MYC] |
| GO:0061614 | miRNA transcription | GO_BiologicalProcess-EBI-UniProt-GOA-ACAP-ARAP_25.05.2022_00h00 | 0,0050214 | [EGR1, MYC] |
| GO:1902895 | positive regulation of miRNA transcription | GO_BiologicalProcess-EBI-UniProt-GOA-ACAP-ARAP_25.05.2022_00h00 | 0,0027183 | [EGR1, MYC] |

**Supplementary Table 3. The results of the KEGG pathway analysis**

Non corrected p values are indicated in the table.

| ID | Term | Ontology Source | p value | Associated Genes |
| --- | --- | --- | --- | --- |
| GO:0044344 | cellular response to fibroblast growth factor stimulus | GO_BiologicalProcess-EBI-UniProt-GOA-ACAP-ARAP_25.05.2022_00h00 | 0,00079 | [EGR3, SPRY4, ZFP36] |
| GO:0048146 | positive regulation of fibroblast proliferation | GO_BiologicalProcess-EBI-UniProt-GOA-ACAP-ARAP_25.05.2022_00h00 | 0,00415 | [MIF, MYC] |
| KEGG:05221 | Acute myeloid leukemia | KEGG_25.05.2022 | 0,00533 | [DUSP6, MYC] |
| KEGG:05222 | Small cell lung cancer | KEGG_25.05.2022 | 0,00983 | [MYC, PTGS2] |
| GO:0032310 | prostaglandin secretion | GO_BiologicalProcess-EBI-UniProt-GOA-ACAP-ARAP_25.05.2022_00h00 | 0,00058 | [MIF, PTGS2] |
| GO:0001516 | prostaglandin biosynthetic process | GO_BiologicalProcess-EBI-UniProt-GOA-ACAP-ARAP_25.05.2022_00h00 | 0,00148 | [MIF, PTGS2] |
| GO:0072203 | cell proliferation involved in metanephros development | GO_BiologicalProcess-EBI-UniProt-GOA-ACAP-ARAP_25.05.2022_00h00 | 0,00014 | [EGR1, MYC] |
| GO:1901724 | positive regulation of cell proliferation involved in kidney development | GO_BiologicalProcess-EBI-UniProt-GOA-ACAP-ARAP_25.05.2022_00h00 | 0,00009 | [EGR1, MYC] |
| GO:0061614 | miRNA transcription | GO_BiologicalProcess-EBI-UniProt-GOA-ACAP-ARAP_25.05.2022_00h00 | 0,00533 | [EGR1, MYC] |
| GO:1902895 | positive regulation of miRNA transcription | GO_BiologicalProcess-EBI-UniProt-GOA-ACAP-ARAP_25.05.2022_00h00 | 0,00288 | [EGR1, MYC] |
